# Long-Term Intermittent Work at High Altitude: Right Heart Functional and Morphological Status and Associated Cardiometabolic Factors

**DOI:** 10.3389/fphys.2018.00248

**Published:** 2018-03-22

**Authors:** Julio Brito, Patricia Siques, Rosario López, Raul Romero, Fabiola León-Velarde, Karen Flores, Nicole Lüneburg, Juliane Hannemann, Rainer H. Böger

**Affiliations:** ^1^Institute of Health Studies, University Arturo Prat, Iquique, Chile; ^2^Department of Preventive Medicine and Public Health, University Autonoma of Madrid, Madrid, Spain; ^3^Department of Biological and Physiological Sciences, Facultad de Ciencias y Filosofía/IIA, University Peruana Cayetano Heredia, Lima, Peru; ^4^Institute of Clinical Pharmacology and Toxicology, University Medical Center Hamburg-Eppendorf, Hamburg, Germany

**Keywords:** high-altitude pulmonary hypertension, chronic intermittent hypobaric hypoxia, altitude, right heart, insulin and ADMA

## Abstract

**Background:** Living at high altitude or with chronic hypoxia implies functional and morphological changes in the right ventricle and pulmonary vasculature with a 10% prevalence of high-altitude pulmonary hypertension (HAPH). The implications of working intermittently (day shifts) at high altitude (hypobaric hypoxia) over the long term are still not well-defined. The aim of this study was to evaluate the right cardiac circuit status along with potentially contributory metabolic variables and distinctive responses after long exposure to the latter condition.

**Methods:** A cross-sectional study of 120 healthy miners working at an altitude of 4,400–4,800 m for over 5 years in 7-day commuting shifts was designed. Echocardiography was performed on day 2 at sea level. Additionally, biomedical and biochemical variables, Lake Louise scores (LLSs), sleep disturbances and physiological variables were measured at altitude and at sea level.

**Results:** The population was 41.8 ± 0.7 years old, with an average of 14 ± 0.5 (range 5–29) years spent at altitude. Most subjects still suffered from mild to moderate symptoms of acute mountain sickness (mild was an LLS of 3–5 points, including cephalea; moderate was LLS of 6–10 points) (38.3%) at the end of day 1 of the shift. Echocardiography showed a 23% mean pulmonary artery pressure (mPAP) >25 mmHg, 9% HAPH (≥30 mmHg), 85% mild increase in right ventricle wall thickness (≥5 mm), 64% mild right ventricle dilation, low pulmonary vascular resistance (PVR) and fairly good ventricle performance. Asymmetric dimethylarginine (ADMA) (OR 8.84 (1.18–66.39); *p* < 0.05) and insulin (OR: 1.11 (1.02–1.20); *p* < 0.05) were associated with elevated mPAP and were defined as a cut-off. Interestingly, the correspondence analysis identified association patterns of several other variables (metabolic, labor, and biomedical) with higher mPAP.

**Conclusions:** Working intermittently at high altitude involves a distinctive pattern. The most relevant and novel characteristics are a greater prevalence of elevated mPAP and HAPH than previously reported at chronic intermittent hypobaric hypoxia (CIHH), which is accompanied by subsequent morphological characteristics. These findings are associated with cardiometabolic factors (insulin and ADMA). However, the functional repercussions seem to be minor or negligible. This research contributes to our understanding and surveillance of this unique model of chronic intermittent high-altitude exposure.

## Introduction

The right cardiac circuit (rather than the left) of high-altitude populations living in chronic hypobaric hypoxia (CH) undergoes major changes. These changes are characterized by elevated pulmonary artery pressure (PAP), right ventricle hypertrophy, and heart and pulmonary vessel remodeling. Some individuals develop high-altitude pulmonary hypertension (HAPH). Individuals who relocate to live permanently at altitude display the same phenomena (Penaloza and Arias-Stella, 2007). A complex series of pathophysiological and physiological mechanisms are responsible for the responses to hypoxia. The first described mechanism is hypoxic pulmonary vasoconstriction (HPV) (von Euler and Liljestrand, 1946), which is followed by several metabolic and molecular alterations, such as an imbalance between endothelial vasoconstrictors and vasodilators, reactive oxygen species (ROS) (Chen et al., 2012), and some associated factors, including insulin and asymmetric dimethylarginine (ADMA) (Richalet and Pichon, 2014; Lüneburg et al., 2016).

Recently, a new type of exposure to altitude has been of interest: long-term chronic intermittent hypobaric hypoxia (CIHH), which has been acknowledged as a distinct pathophysiological condition, including higher blood pressure at altitude and acute mountain sickness persistence on day 1 (Richalet et al., 2002; Brito et al., 2007). This exposure implies long-term exposure to shifts of 4 to 15 days at an altitude above 3,500 m, which are followed by a resting period of the same number of days at sea level (Richalet et al., 2002; West, 2002). Mining, observatory, army, and frontier control personnel are frequently exposed to these conditions, and their numbers are dramatically increasing. In Chile alone, it has been estimated that there are over 65,000 workers exposed to this condition (Brito et al., 2007). Therefore, many aspects of the underlying molecular mechanisms and clinical consequences are not well-known. Some studies in humans have demonstrated changes in the right heart circulation that are similar to those occurring in CH, such as a rise in PAP and right ventricular enlargement and/or hypertrophy (Richalet et al., 2002; Sarybaev et al., 2003; Brito et al., 2007). Remodeling of pulmonary vessels has also been demonstrated in animal models (Brito et al., 2015). Notwithstanding, there is scarce research assessing these changes over long durations in larger groups of individuals or looking for new potential associations with other physiological and biochemical variables as associated factors.

The endothelium has been known to play an important role in the regulation of systemic and pulmonary vascular tone, which mainly occurs by secreting the potent vasodilator nitric oxide (NO). NO is synthesized by endothelial NO synthase (NOS), which is competitively inhibited by the endogenous compound asymmetric dimethylarginine ADMA (Böger, 2006). In contrast to ADMA, symmetric dimethylarginine (SDMA) does not directly interfere with NOS activity. Elevated levels of ADMA are a cause of vasoconstriction and high blood pressure and have been associated with a high risk of cardiovascular events and mortality (Zoccali et al., 2001; Böger et al., 2009a,b).

Animal research has already provided some information about molecular or functional interactions in CIHH. Interestingly, NO bioavailability and ROS production have been demonstrated to play a major role in vascular adaptation to altitude hypoxia (Siques et al., 2014b; Lüneburg et al., 2016; Waypa et al., 2016). The morphological, physiological and molecular changes appear to be similar to those occurring in CH, but they may be less pronounced in CIHH (Brito et al., 2008; Siques et al., 2014b; Lüneburg et al., 2016).

Thus, it was expected that altered right heart circuit status and possible associated factors (metabolic, labor, and physiological) would be found in people undergoing long intermittent work at high altitude. Therefore, a cross-sectional study was performed with the aim of determining the morphological and functional status of the right cardiac circuit in miners working intermittently at an altitude between 4,400 and 4,800 m for a period of more than 5 years. Moreover, the study assessed several physiological and metabolic factors to determine whether they were associated with cardiac parameters and whether some of these featured a distinctive response in the evaluated condition.

## Materials and methods

### Subjects and study design

A cross-sectional study was performed in a random sample of 120 healthy native Chilean male miners working in a mine settlement in the northern part of Chile at an altitude of 4,400 or 4,800 m (53 and 47% of the study population, respectively) in a shift regimen (7 days at altitude followed by a resting period of 7 days at sea level). The miners slept at 3,800 m and worked a 12-h day shift at highest altitude, with the trip from the dormitories to the pit lasting 30 min. All subjects had undergone a medical examination and laboratory tests to determine altitude fitness. The inclusion criteria were working in shifts (7X7) at high altitude (above 4,000 m) for more than 5 years and a healthy status without serious comorbidities. The exclusion criteria were diabetes, hypertension, diagnosed obstructive sleep apnea, supplementary oxygen in the dormitories and any cardiopulmonary disease.

Written informed consent was obtained from all participants in accordance with the Declaration of Helsinki. The study was approved by the Research Ethics Committee of Universidad Arturo Prat, Iquique, Chile.

### Measured variables

Measures were taken (1) at altitude (at the mine's health facility) early in the morning 18 h after arrival (after one night's sleep) and (2) at sea level (SL) at an ambulatory medical facility in Iquique, Chile during day 2 of the resting period. An exact definition of the measurement time of each variable is provided below.

### General data

Age, weight, height, body mass index (BMI), calculated as weight (kg) divided by height squared (m^2^), waist perimeter (WP), smoking habit, years at altitude, physical activity and medical status were assessed during the basal screening at SL.

### Physiological parameters

Systolic blood pressure (SBP), diastolic blood pressure (DBP), heart rate (HR), and hemoglobin oxygen saturation (SaO2) were determined at each study time point (at altitude and SL) in the morning. SBP and DBP were measured in the right arm of each participant while seated and after 5 min of rest using appropriately sized cuffs and calibrated standard mercury sphygmomanometers according to international guidelines (Chobanian et al., 2003; European Society of Hypertension-European Society of Cardiology Guidelines Committee, 2003). Heart rate was measured with an HR-100C Omron device (Omron, Health Care Inc.®, Bethesda, Maryland; USA), and SaO2 was determined using a finger pulse oximeter (POX050, Mediaid®, Cerritos, CA; USA). The mean of two measurements separated by a 5-min interval was taken as a valid determination of BP, HR, and SaO2. Same measures were obtained at SL for comparison.

### Acute mountain sickness (AMS) and sleep measurements

The Lake Louise Score-AMS self-assessment test (LLS; Roach et al., 1993), validated in similar Chilean populations (Richalet et al., 2002; Brito et al., 2007), was performed at altitude 18 h after arrival, including the first night's sleep. AMS was diagnosed when headache and at least one other symptom occurred and a LLSs of ≥3 was reached. Severity was assessed according to the following categories: mild (3–4), moderate (5–10), and severe (11–15) (Hackett, 2003). The modified Spiegel questionnaire, validated in similar populations (Richalet et al., 2002; Brito et al., 2007), was recorded to assess sleep status at the same time. Both scores were obtained at SL for comparison.

### Hematological and biochemical measurements

Blood samples were taken at SL in the morning after 8 h of fasting through venous puncture without stasis: hematocrit (Htc), hemoglobin (Hb), lipid profile, glycemia, and insulin. Additionally, insulin sensitivity or resistance [homeostatic model assessment (HOMA-IR) index] was calculated by HOMA program V.2.2 (Diabetes trial unit, University of Oxford). The biomarkers asymmetric dimethylarginine (ADMA) and symmetric dimethylarginine (SDMA) were measured using a validated liquid chromatographic–tandem mass spectrometric assay as described previously (Schwedhelm, 2005). The ADMA reference range is ≤0.732 μmol/L, which was derived from a large population-based cohort (Schwedhelm et al., 2009). An SDMA reference range of ≤0.53 μmol/L was later established by the same author (Schwedhelm et al., 2011).

### Echocardiographic assessment

An echocardiographic assessment was performed by two experienced cardiologists at SL facilities, in the morning after a 1 h rest, using an echocardiograph (GE Vivid-I®, GE Healthcare Systems, Tirat Carmel, Israel) with a 1.5–3.6 MHz phased array probe. Left ventricular end diastolic and end systolic measurements and left ventricular septal and posterior wall thicknesses were obtained from parasternal long axis view in M-mode with the ultrasound beam aligned to tips of mitral leaflets. Left ventricular ejection fraction (LVEF) was calculated from the M-mode recordings using Teichholtz formula (Teichholz et al., 1976). Right heart measurements were obtained after aligning the tip of ultrasound beam at true left ventricular apex. Pulmonary ejection time and pulmonary acceleration times were obtained with pulse Doppler recordings of the pulmonary valve from a parasternal short axis view at aortic valve level. Mean pulmonary artery pressure (mPAP) was calculated from pulmonary acceleration time according to Mahan formulas (Dabestani et al., 1987). Tricuspid annular systolic excursion was measured in M-mode from an apical four-chamber view with the ultrasound beam aligned to the lateral aspect of the tricuspid annulus. Right ventricular free wall thickness (RVWT) was obtained from a subcostal four-chamber view. Tricuspid annular plane systolic excursion (TAPSE index) was used for right ventricle (RV) performance (Kaul et al., 1984; López et al., 2012). Pulmonary vascular resistance (PVR) was calculated according to the formula described by Abbas et al. (2003). Similarly, other authors have noted that echocardiography for right heart assessment, including pulmonary hypertension, has shown a high correlation (*r*^2^) with invasive right heart catheterization (Kojonazarov et al., 2007; Taleb et al., 2013). All measurements and reference values were acquired according to American Society of Echocardiography Guidelines (Rudski et al., 2010). Two separate cut-off criteria were studied to define pulmonary hypertension (PH): (a) HAPH's consensus (León-Velarde et al., 2005), which is defined at mPAP ≥30 mmHg, and (b) SL PH's criteria (Rubin and American College of Chest Physicians, 2004), which is defined at mPAP ≥25 mmHg. The reason for including the SL cut-off was two-fold: (1) because the echocardiogram was performed at SL and (2) to have a comparative panorama since the current condition under examination entailed a substantial period of both high-altitude and SL exposure.

### Data analysis

All data were entered into a database and analyzed using IBM SPSS, V21.0® statistical package (Armonk, NY, USA). For qualitative variables, absolute and relative frequencies were calculated. For quantitative variables, proportions, means, standard deviations, and standard errors were calculated. Normality of the distribution was checked using Kolmogorov–Smirnov test. All variables, except for LLS, were normally distributed. Therefore, a non-parametric Wilcoxon Test was used for LLS. Student's *t*-test for related or independent samples was used as appropriate. Pearson's chi-square test was used for proportional differences for independent variables. Additionally, Pearson's correlation was performed between quantitative variables. Binary logistic regression models were used to assess the association of all variables measured with the mPAP using two different cut-offs (<30 vs. ≥30 mmHg and <25 vs. ≥25 mmHg). After univariate analyses of all variables, the statistically significant variables were introduced into a multivariable logistic regression model using the forward stepwise method. The results were presented as crude (mutually adjusted) odds ratios (OR) and 95% confidence intervals (CI). The significance level was established at *p* < 0.05.

To look for association patterns, a multiple correspondence analysis was performed between categorical variables, which might display a more comprehensive panorama. Biomedical, occupational and metabolic variables were chosen and dichotomized at their normal values. The graph and variances of each dimension are provided. Angles <60° are considered as association and the longer distance of the variable from its origin the better represented. A Cronbach's alpha of >0.50 was considered appropriate.

## Results

### General characteristics

The study group was 120 miners with a mean age of 41.8 ± 0.7 years and a mean exposure to CIHH of 14 ± 0.5 years. Most study participants were overweight (BMI: 26.3 ± 0.3 kg/m^2^) and sedentary (<3 weekly <30 min moderate exercise sessions, according to Chilean criteria) (MINSAL Ministerio de Salud, 2006), and a third of them were current smokers. Table 1 gives a complete overview of the demographic and anthropometric characteristics of the study group.

**Table 1:** General characteristics of chronic intermittent hypoxia group.

**Variables**	**$\bar{X}$ ± SE; (range)**	**%**
Age (years old)	41.8 ± 0.7 (20–58)	
<40		40.8
≥40		59.2
Years at altitude	13.9 ± 0.5 (05–29)	
5–<10		20.8
≥10		79.2
Altitude of work (m)	4.600 ± 0.2 (4.400–4.800)	
4,400		53.3
4,800		46.7
BMI (kg/m^2^)	26.3 ± 0.3 (16.6–34.9)	
<25		36.7
≥25–<29.9		52.5
≥30		10.8
Waist Perimeter (cm)	97.1 ± 09 (61–121)	
≤100		67.5
>100		32.5
Smoking Status		
Yes		34.2
No		65.8
Sedentary		
Yes		80.2
No		19.2

*Values for quantitative variables are means ($\bar{X}$) ± standard error (SE) and ranges; values for qualitative variables are proportions (%)*.

### Physiological parameters

As expected, both SBP and DBP were higher at altitude than at SL, along with a slight increase in HR. Approximately 40% of the subjects had elevated SBP at altitude (≥130 mmHg, *p* < 0.01), and 18% had elevated DBP (≥90 mmHg, *p* < 0.05). SaO2 differed between SL and altitude in proportion to the level of altitude. However, 30% of the study population had SaO2 levels ≤88% at altitude (Table 2).

**Table 2:** Physiological parameters.

**Variables**	**$\bar{X}$ +SE**	**$\bar{X}$ +SE**	***p***
	**SL**	**Altitude**	
SBP	109.4 ± 1.0	126.2 ± 1.0	<0.001
DBP	69.9 ± 0.9	81.0 ± 0.7	<0.001
HR	71.5 ± 1.1	82.0 ± 1.0	<0.001
SaO2	97.7 ± 0.1	89.6 ± 0.3	<0.001
LL	0.61 ± 0.1	2.7 ± 0.2	<0.001
Spiegel test	10.6 ± 0.3	14.5 ± 0.4	<0.001

*Systolic blood pressure (SBP; mmHg), diastolic blood pressure (DBP; mmHg), heart rate (HR; b/m), hemoglobin oxygen saturation (SaO2; %), Lake Louise (score) and Spiegel test (score), at sea level (SL) and at altitude. Values are means ($\bar{X}$)±SE (standard error), and p was obtained from t-test of related samples*.

### AMS and sleep disturbances

Despite long exposure to high altitude, a rather high proportion of individuals showed AMS (38.4%) on the first day, which was mainly moderate, and remarkably many study participants had cephalea (47.5%). Most individuals declared that they had mainly regular non-satisfactory sleep (75%), whereas the sleep disturbances and AMS measured at SL were minimal (20% and 12.5%, respectively). The presence of AMS was significantly associated with mPAP ≥25 and mPAP ≥30 mmHg (*p* < 0.05).

### Hematocrit and hemoglobin

Mean Htc and Hb were almost within the normal range (70% below 17 mg/dL). Only two individuals had Hb values of 19 mg/dL, and none had Hb above 21 mg/dL (Table 3).

**Table 3:** Hematological and biochemical measurements at sea level.

**Variables**	**$\bar{X}\pm$ SE**	**Reference range**
Hematocrit (%)	47.6 ± 0.3	41–49
Hemoglobin (mg/dL)	16.2 ± 0.1	13.0–17.5
Total cholesterol (mg/dL)	193.1 ± 3.3	50–200
HDL-cholesterol (mg/dL)	43.3 ± 0.8	40–60
LDL-cholesterol (mg/dL)	114.5 ± 2.8	80–125
VLDL-cholesterol (mg/dL)	35.3 ± 1.8	10–35
Triglycerides (mg/dL)	175.9 ± 8.9	30–150
Total cholesterol/ HDL-cholesterol	4.5 ± 0.1	3
LDL-cholesterol/HDL-cholesterol	2.8 ± 0.9	3
Glycemia (mg/dL)	89.4 ± 1.3	90–110
Insulinemia (IU)	11.4 ± 0.6	10–20
Homeostatic model assessment (HOMA-IR)	1.4 ± 0.8	2.6
Asymmetric dimethylarginine (ADMA; μmol/L)	0.83 ± 0.2	0.73
Symmetric dimethylarginine (SDMA; μmol/L)	0.54 ± 0.2	0.53

*Values are means ($\bar{X}$) ±standard error (SE) and reference range*.

### Lipid profile

In 48% of the individuals, triglycerides were elevated above 150 mg/dL, and mean triglycerides and VLDL-cholesterol was also elevated. Although mean total cholesterol was within the normal range, 42% of the study participants had values over 200 mg/dL. Mean HDL-cholesterol and LDL-cholesterol were within the respective normal ranges. The Castelli index (total cholesterol and HDL-cholesterol) was within its upper limit, and HDL/LDL index was normal (Table 3), although 20% of subjects had low HDL, and 30% had elevated LDL.

### Glycemia, insulin, and HOMA-IR

Mean glycemia and insulin values were within normal ranges. The mean HOMA-IR index was also normal (Table 3). However, for subjects with insulin above 20 IU (11%), the HOMA-IR index was 3.3 ± 0.2. Insulin was found to significantly correlate (*p* < 0.01) with BMI (*r* = 0.34); WP (*r* = 0.39), SBP at altitude (*r* = 0.25), VLDL and triglycerides (TG) (both *r* = 0.25) and mPAP (*r* = 0.22; *p* < 0.05).

**Figure 1:**
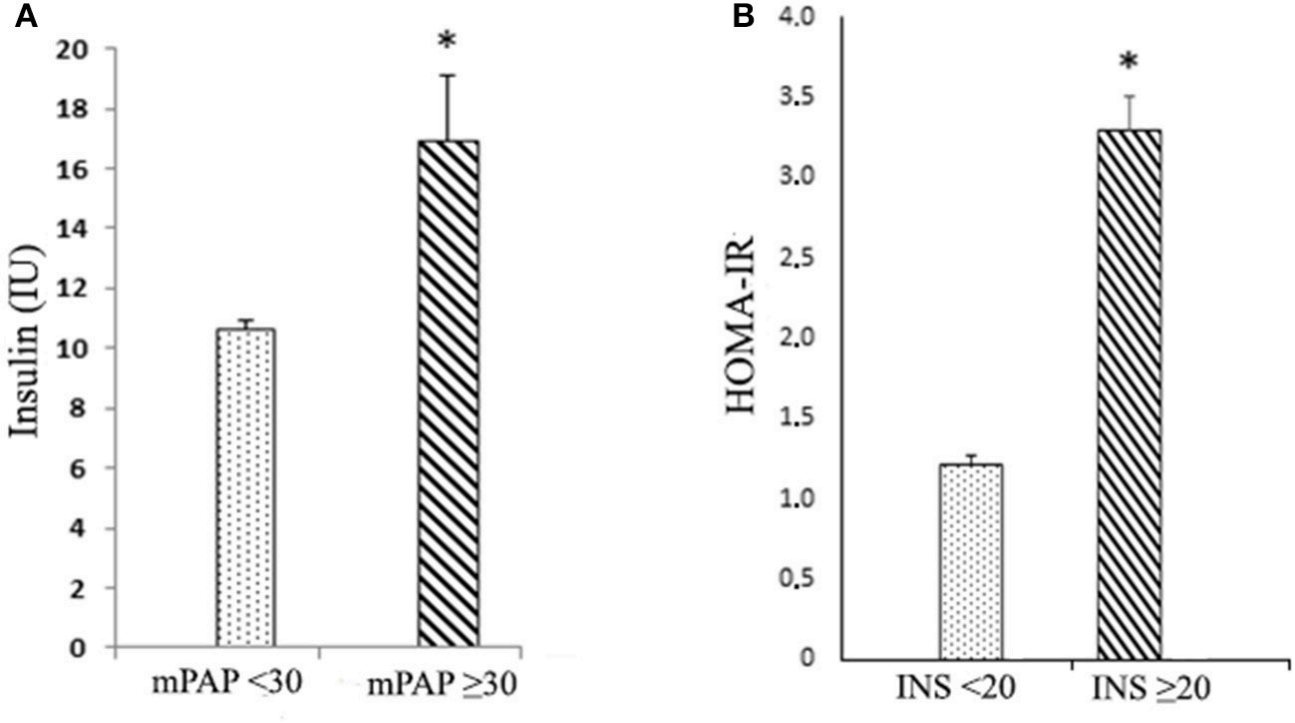
Comparison between insulin, mPAP, and HOMA-IR **(A)** insulin (INS; IU) according to mean pulmonary artery pressure values (mPAP; mmHg); cut-off point < ≥30 mmHg; and **(B)** homeostatic model assessment (HOMA-IR; Index) according to insulin values: cut-off point < ≥20 IU. Values are means ($\bar{X}$)±SE (standard error); *p < 0.01.

Upon further analysis of the association of mean insulin levels with mPAP values, a distinctive difference in insulin concentrations at each cut-off point of mPAP was found. At mPAP ≥30 mmHg, mean insulin was 16.9 ± 2.14 IU, whereas at mPAP <30 mmHg, a lower mean insulin value was observed (10.6 ± 0.32 IU; *p* < 0.01, Figure 1A). Those with high insulin levels had a higher HOMA-IR (Figure 1B). Moreover, this association was further corroborated by the correlation of HOMA-IR with higher mPAP (*r* = 0.24, *p* < 0.001).

### ADMA

**Figure 2:**
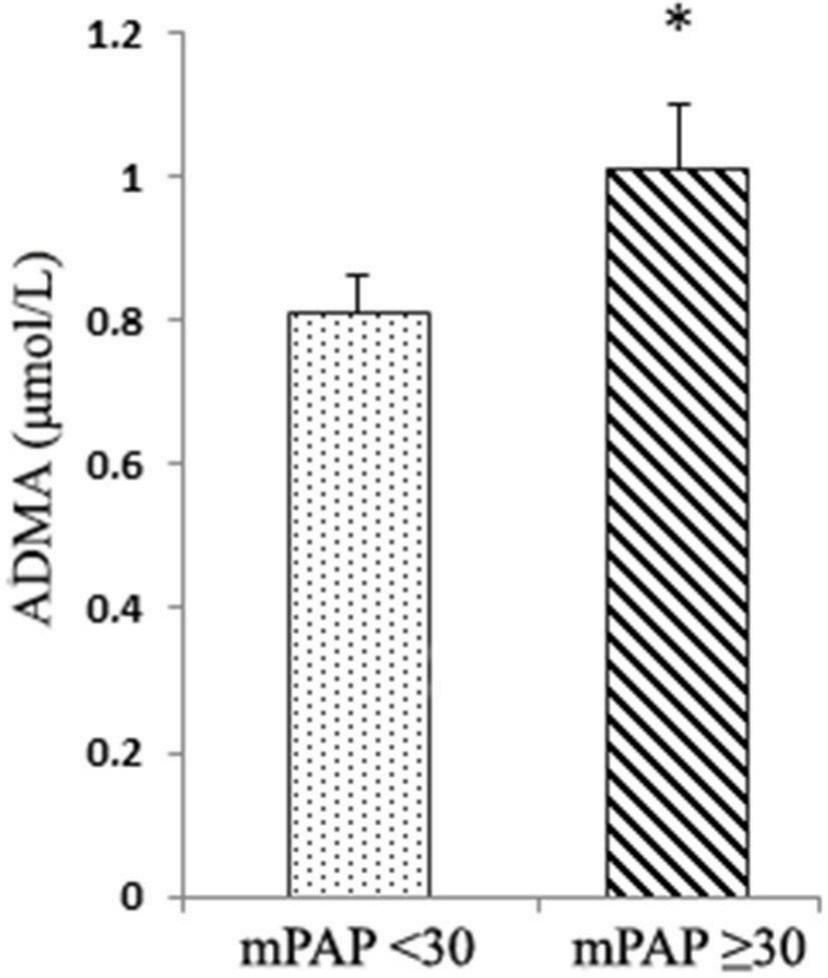
Comparison of ADMA and mPAP (cut-off point < ≥30 mmHg). Asymmetric dimethylarginine (ADMA; μmol/L); mean pulmonary artery pressures (mPAP; mmHg). Values are means ($\bar{X}$)±SE (standard error); *p < 0.01.

Mean ADMA concentration was slightly elevated (0.83 ± 0.2 μmol/L) compared to the reference range (Table 3). Half of the subjects had ADMA concentrations ≥0.80 μmol/L. ADMA was positively correlated (*p* < 0.05) with WP (*r* = 0.21), Hb (*r* = 0.20), and SDMA (*r* = 0.82; *p* < 0.001). Most importantly, ADMA was correlated with mPAP (*r* = 0.21; *p* < 0.05) and RVWT (*r* = 0.30; *p* < 0.001). Distinctly, mean ADMA concentration was 1.01 ± 0.15 μmol/L in subjects with mPAP ≥30 mmHg, as opposed to 0.81 ± 0.18 μmol/L in subjects with mPAP <30 mmHg (*p* < 0.001; Figure 2).

### Echocardiographic findings (mPAP and morphological status)

Most individuals had mPAP within normal ranges (73.9%) with wide variability. Nevertheless, 26.1% of the subjects had elevated values at a cut-off point of 25 mmHg, but only 9.2% could be categorized as truly HAPH (≥30 mmHg), and none exceeded an mPAP of 36 mmHg (Table 4).

**Table 4:** Echocardiographic findings at sea level.

**Variables**	**$\bar{X}+$ SE**	**Range**	**SL reference value**	**Out of reference value (%)**
SPAP (mmHg)	27.6 ± 0.5	13.0–38.0	30	36
mPAP (mmHg) Mahan	20.2 ± 0.6	10.2–35.8	<25	73.9
≥25 overall	–		–	26.1
≥25–<30	–		–	16.9
≥30	–		–	9.2
RVWT (mm)	6.3 ± 0.1	4.0–10.0	<5	85
<5 mm				15
≥5 mm				85
FCRV (mm)	30.8 ± 0.4	23.0–43.0	<28	64
RVOT short axis (mm)	29.6 ± 0.4	23.0–39.0	27-30	45
<30 mm				55
≥30 mm				45
RAA (cm^2^)	15.1 ± 0.2	10.0–24.0	<18	15
PVR (Wood units; 240 din/cm*s^2^)	1.08 ± 0.02	0.59–1.76	<1.5	4.8
LVEF (%)	70.1 ± 0.7	50.0–87.0	≥56	4.2
AD (mm)	30.5 ± 0.3	21.0–41.0	<34	22.6
TAPSE index (cm)	2.3 ± 0.3	1.25–2.90	≥1.6	0.9

*Systolic pulmonary artery pressure (SPAP), mean pulmonary artery pressure by Mahan (mPAP, right ventricle wall thickness (RVWT, four-chamber right ventricle (FCRV), right ventricle outflow track (RVOT), right atrium area (RAA), pulmonary vascular resistance (PVR), left ventricle ejection fraction (LVEF), aortic diameter (AD) and tricuspid annular plane systolic excursion (TAPSE). Values are means ($\bar{X}$) ± standard error (SE), range, sea level (SL) cut-off values (reference: American Society of Echocardiography; Rudski et al., 2010) and proportions out of reference value (%)*.

**Figure 3:**
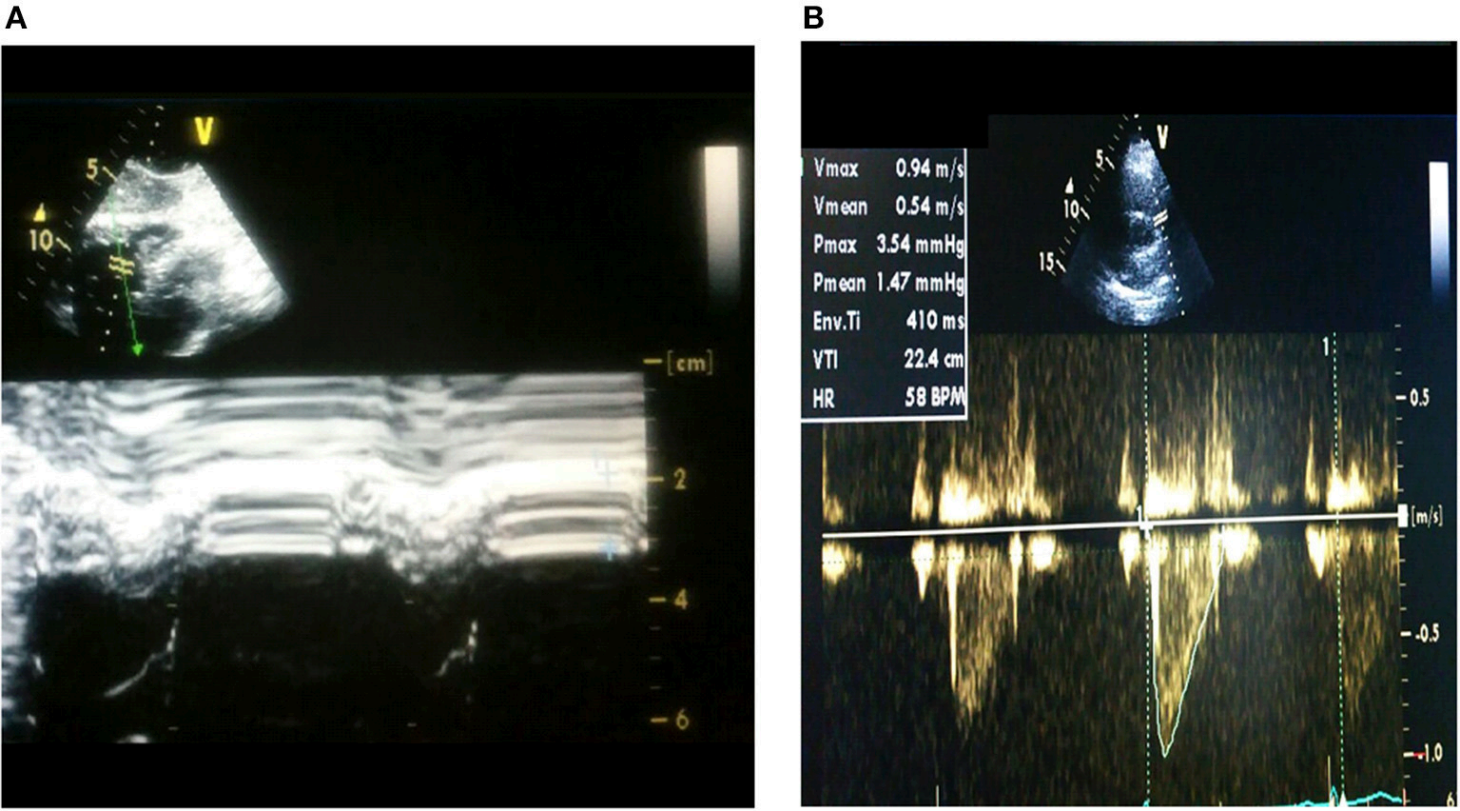
Representative echocardiographic images **(A)** right ventricular hypertrophy and **(B)** Acceleration curve of pulmonary flow at pulmonary artery outflow tract.

Regarding morphological status, it was noted that 85% of the individuals had mild right ventricle hypertrophy (RVH), and over half showed a grade of right ventricular (RV) dilation supported by an increase in FCVR and right ventricle outflow track (RVOT) values. However, a minimal percentage (15%) of right atrium enlargement is seen (Table 4). A representative figure of RVH is shown in Figure 3A, and pulmonary acceleration curve at outflow tract in Figure 3B.

Most subjects display PVR within the normal range, except for 4.8% of the individuals, who have slightly increased PVR. Despite the latter finding, the subjects with mPAP ≥30 mmHg had a value of 1.33 Wood units vs. 1.04 Wood units with mPAP <30 mmHg (*p* < 0.001). The RV performance index (TAPSE) shows no impairment and good performance in this group. For the left ventricle, the left ventricle ejection fraction is within the normal range and the aortic diameter is only mildly enlarged in 22.6% of the individuals (Table 4).

Some significantly positive correlations (*p* < 0.01) were found in all correlations performed between echocardiographic variables: (a) mPAP vs. RVWT *r* = 0.39, RVOT *r* = 0.35, and PVR *r* = 0.40; (b) RVWT vs. RVOT *r* = 0.30 and PVR *r* = 0.22; and (c) right atrium area (RAA) vs. four-chamber right ventricle (FCRV) *r* = 0.44 and PVR *r* = 0.21.

However, only two associations were shown when both cut-off values for mPAP (25 and 30 mmHg) were used in the univariate logistic regression and in the forward stepwise logistic multivariate regression final model. At the cut-off value of 25 mmHg, ADMA (OR 8.84; CI 1.18–66.39, *p* < 0.05) and insulin (OR: 1.07, CI 1.01–1.13, *p* < 0.05) showed an association. At the cut-off value of 30 mmHg, the same associations were observed: ADMA (OR: 10.74, CI 1.16–99.9, *p* < 0.05) and insulin (OR: 1.11, CI 1.02–1.20, *p* < 0.05). All OR were adjusted by smoking status, age, and BMI.

### Multiple correspondence analysis

Because only two associations were found, a multiple correspondence analysis was performed. This statistical tool allows visualize association patterns or profiles between different dichotomized variables to be determined: occupational and biomedical (years old and years at altitude), metabolic (ADMA, insulin, triglycerides, BMI, and waist perimeter) and echocardiographic (right ventricle wall thickness and mPAP) were introduced. To interpret the graphical representation, category associations can be detected by their proximity whose reference is the angle formed with the coordinates origin (small angles between two categories indicates greater association) and the relative distance to the origin.

**Figure 4:**
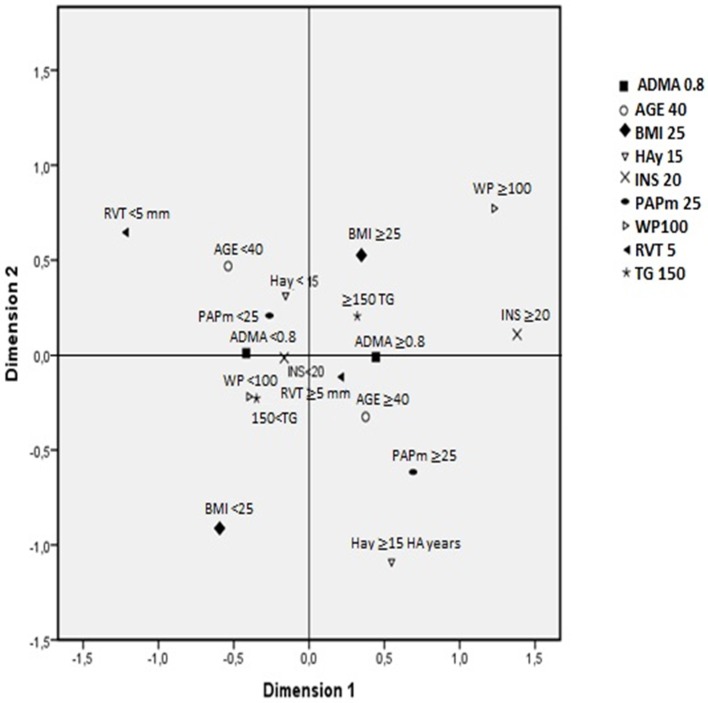
Association patterns between variables by multiple correspondence analysis. Variables and cut-off points: asymmetric dimethylarginine (ADMA; μmol/L; < ≥0.8), age (years old; < ≥40), body mass index (BMI; kg/ m^2^; < ≥25), high-altitude years (HAy; < ≥15 years), insulin (INS; IU; < ≥20), mean pulmonary artery pressure (PAPm, mmHg; < ≥25), waist perimeter (WP; cm; < ≥100), right ventricle wall thickness (RVT; mm; < ≥5) and triglycerides (TG; mg/dL; < ≥150). Explained proportion of total variance of dimension 1 = 57.3% and dimension 2 = 42%. Cronbach's alpha: 0.594.

Therefore, on one hand, two different profiles are shown. In one profile, metabolic variables were within normal values, and age <40 and years at altitude <15 were associated with mPAP <25. In the other profile, altered metabolic values, higher age, and over 15 years at altitude were found to be associated with mPAP ≥ 25. On the other hand, that mPAP > 25 and > 15HAyears are strongly associated (small angle), mPAP >25 and insulin > 20 are moderately associated (medium angle) and mPAP > 25 and age <40 (180 degree angle) suggest opposite directions (no association) are depicted (Figure 4). Interestingly, when the same procedure was performed with mPAP ≥ 30, the results were similar.

## Discussion

This cross-sectional study, in long-term CIHH working shifts at high altitude, has the following main findings: (1) a distinctive and unique pattern of physiological responses was determined, wherein a third of subjects showed moderate AMS persistence; (2) high proportions of elevated mPAP (26.1%) and HAPH (9.2%) were found by echocardiography; and (3) specific cardiometabolic variables (high triglycerides, insulin resistance, high ADMA, increased waist perimeter and BMI) appeared to be associated factors, with insulin and ADMA clearly associated with elevated mPAP.

### General aspects

This cross-sectional study with a large number of subjects working intermittently in a long-term CIHH at high altitude contributes three important concepts (mentioned above), which will be individually discussed for methodological reasons; however, these concepts appear to be related and intertwined as a whole.

The first concept is that this population seems to share distinctive and unique features. In fact, there is a remarkable proportion of overweight and sedentarism, as has been previously reported (Esenamanova et al., 2014). Smoking habit proportion is similar to Chilean prevalence. Physiological responses are also coincident with previous reports in that BP rises at altitude but reaches normal values at SL. Moreover, it has been described that BP decreases after day 2 during the shift at high altitude, but it does not reach SL values (Richalet et al., 2002; Brito et al., 2007); nonetheless, a certain proportion of individuals maintain an SBP and DBP within a rather elevated range at altitude according to previous reports (Siques et al., 2009). Conversely, SaO2 drops at altitude and is fully recovered at SL, and the mean SaO2 reached at altitude is similar to acclimatized subjects; however, almost one-third of the subjects have lower SaO2 values, which could be explained by individual variability or a poor response to altitude (day 1's impact) (Brito et al., 2007). Additionally, coincident with previous reports in humans, Htc and Hb do not show pathological values or excessive erythrocytosis (Richalet et al., 2002; Brito et al., 2007; Siques et al., 2007). The latter adds support to the suggestion that under this regimen, high or excessive erythrocytosis is rare. Likewise, consistently high AMS presence (mostly moderate), cephalea and sleep disturbances despite the extensive elapsed time are additional distinctive features. These findings are also coincident with previous studies in which AMS was present during the first 1 or 2 days of a shift at high altitude (Richalet et al., 2002; Brito et al., 2007). However, some cohort studies have described a decline during following exposures (Richalet et al., 2002; Wu et al., 2009). The reasons are beyond the scope of this study, but may be due to an inability to acclimatize properly, the loss of acclimatization during the shift at SL or rapid ascent.

### Metabolic responses

#### Lipid profile: TG and total cholesterol (T-Chol)

Another relevant result of this study are the changes in lipid profiles. It has been assumed by several authors that under CH or CIHH, individuals are prone to good metabolic features (Anderson and Honigman, 2011; Ezzati et al., 2012). However, recent evidence has shown that there is an increasing proportion of individuals with marked metabolic alteration (Mohanna et al., 2006), which would be related to chronic mountain sickness (Miele et al., 2016) and other altitude diseases (San Martin et al., 2017). In fact, our results support this observation; in this model of exposure, a rather high proportion (almost half of the subjects) displayed an altered lipid profile. Previous studies have consistently reported an increase in TG and contradictory results for T-Chol (Li et al., 2007; Siques et al., 2007). The most remarkable changes seen in this study are an increase in TG, VLDL and T-Chol, but the mean Castelli index remains at its upper limit.

Hypoxemia may disturb lipid metabolism by upregulating hepatic SCD-1, leading to *de novo* TG synthesis, an increase of adipose tissue lipolysis, lipoprotein secretion and decrease of lipoprotein clearance (Li et al., 2007; Drager et al., 2012; Siques et al., 2014a). Thus, it could be surmised that TG alteration could be another distinctive feature of exposure to CIHH and may be a matter of concern. Recognition of TG alteration as a cardiovascular risk factor is a growing issue (Assmann et al., 1996).

### Insulin, insulin resistance (IR)

Complementary to the above metabolic findings, insulin and IR have been noted as tightly related to the development of PH and could be considered associated factors (Zamanian et al., 2009). Their relation to HAPH is still to be demonstrated. However, the finding that the insulin level correlated to anthropometric variables (BMI and WP) and to mPAP—and this population is mostly overweight—might suggest a similar role in HAPH as has been reported either for IR (McLaughlin and Rich, 2004) and/or PH (Taraseviciute and Voelkel, 2006) at SL. The findings of a cut-off point of different insulin values according to mPAP and that higher insulin values are also associated with higher mPAP further support insulin's role. IR may also share other pathophysiological conditions that are present under hypoxia, such as elevation of cytokines (interleukin-6, monocyte chemotactic protein) and ADMA (Zamanian et al., 2009). Recently, an inverse relationship between oxygen hemoglobin saturation and IR has been described in chronic hypoxia. There are many physiopathological explanations (Miele et al., 2016), including the disruption of leptin pathways (Polotsky et al., 2003).

### ADMA as a marker of cardiometabolic risk

ADMA is a competitive NOS inhibitor that has been identified as a regulator of NO production (Böger, 2006). ADMA is endogenously present in the human body and inhibits NO-dependent vasodilation *in vitro* (Vallance et al., 1992) and *in vivo* (Böger et al., 1998). ADMA is degraded by dimethylarginine dimethylaminohydrolase (DDAH). Disruption of the ADMA/DDAH pathway causes endothelial dysfunction and elevated blood pressure in the systemic and pulmonary circulation (Leiper et al., 2007). Therefore, the possible role of ADMA in HAPH is a focus of current research. The current results show a significant elevation of ADMA at altitude, and for the first time, they show a correlation of ADMA with both mPAP and right ventricle wall thickness, suggesting a pivotal pathophysiological role of this pathway in the development of pulmonary vascular dysfunction at high altitude. Moreover, a clear cut-off value of ADMA was observed according to mPAP.

Recently published data support the current findings. In rats subjected to long-term CIHH, an impaired NO pathway secondary to elevated ADMA and increased ROS were found (Siques et al., 2014b; Lüneburg et al., 2016). Furthermore, young healthy adults first exposed to CIHH develop elevated ADMA concentrations over the time (Lüneburg et al., 2017). Therefore, ADMA may be a useful biomarker for subjects with HAPH.

### PAP and right heart status

An increase in PAP is a well-known physiological response to hypoxia (von Euler and Liljestrand, 1946). Additionally, permanent residents have changes in their pulmonary vascular circuit that might account for a prevalence up to 10% of HAPH (León-Velarde et al., 2005; Penaloza and Arias-Stella, 2007). Unfortunately, most information comes from acute or chronic exposure and rarely from long-term CIHH; therefore, the described changes might not be entirely applicable to CIHH. Research conducted under different commuting exposure regimes in humans (2-year cohort, 30 × 30 shift; 2-year cohort, 7 × 7 shift) and in a 12-year cross-sectional study (5 × 2 shift) at altitudes between 3,550 and 4,400 m) also described an increase in mPAP, right ventricle enlargement or ventricle hypertrophy, although a small number of subjects was included (Richalet et al., 2002; Sarybaev et al., 2003; Brito et al., 2007). The latter author determined a 4% prevalence of HAPH in CIHH at 3,550 m. Interestingly, experimental studies in rats led to the suggestion that RV changes were achieved to a lesser extent in CIHH than in CH (Corno et al., 2004; Brito et al., 2008).

Despite several limitations (cross-sectional study, echocardiogram performed at SL, only clinical functional capacity assessment and previously selected subjects for high-altitude work) of the current study, the results showed a strikingly high proportion of mPAP over 25 mmHg with a proportion of HAPH greater than previously reported that is similar to the prevalence in CH. Likewise, it could well be inferred that subsequent pulmonary vasculature remodeling triggered by HPV was present, as reported in CH (Penaloza and Arias-Stella, 2007; Sylvester et al., 2012) and reproduced in rats under long-term CIHH (Brito et al., 2015). Similarly, in light of the results and despite the concept of “turn on-turn off” biological responses in this condition (Powell and Garcia, 2000), it seems reasonable to infer that in the current model, individuals undertake a more prolonged pulmonary hypertensive state than expected in this condition at high altitude.

Moreover, the HAPH levels found in this study could be considered mild, which is supported by the fact that no subjects had mPAP values over 36 mmHg and that the subjects had a current healthy status without claims of functional capacity impairment. The lack of functional repercussions (Penaloza et al., 1963) and a mild or moderate HAPH have been described for CH and acute hypoxia (Naeije and Dedobbeleer, 2013); this phenomenon has been noted as the paradox of HAPH (Grover, 2014). Nevertheless, some disputes still exist regarding exercise capacity at high altitude and HAPH. In fact, a recent study of Kyrgyz highlanders with HAPH found a mild reduction in exercise performance and reduced quality of life (Latshang et al., 2017). Accordingly, these rather contradictory findings have led to introduction of the concept of “pulmonary vascular reserve” as a complex mechanism that determines good or poor exercise capacity (Groepenhoff et al., 2012; Naeije and Dedobbeleer, 2013; Pavelescu et al., 2013).

Likewise, this study also shows a striking proportion of RVWT enlargement, suggesting that in the long term, almost all subjects experience RV remodeling and that an mPAP value of 25 mmHg would be sufficient to generate significant changes in the right heart. Whether this is merely an acclimatization response in this model of exposure that causes the RV to increase its performance as a consequence of its homeometric adaptation to afterload increase (Kolár and Ostádal, 1991; Naeije and Dedobbeleer, 2013; Richalet and Pichon, 2014) and/or ROS activation by hypoxia of AMP kinase proteins (Chen et al., 2012; Waypa et al., 2016) or other mechanisms remains to be elucidated. Additionally, a vascular hyperdynamic state triggered by adrenergic activation and autonomic system imbalance must be considered for exerting its influence on the above variables and possibly in RV dilation (Richalet and Pichon, 2014). Conversely, right atrium morphological changes account for a very low proportion. Complementary, several weak correlations between RV morphological parameters showed linearity with RV afterload (mPAP value). These findings support the consistency of the measurements, and they are in line with the PH evolving process (ACCF/AHA, 2009).

Further analysis of the PVR results is limited since the indirect method of measurement could bias their interpretation and is also controversial. In fact, although some studies mentioned above support a high correlation with right heart catheterization, some others have described this method as accurate but with only moderate precision (Naeije, 2003; Rudski et al., 2010; Naeije and Dedobbeleer, 2013). If these results were accurate, it would support a more hyperdynamic state and milder surmised vascular remodeling, most likely as the result of greater NO bioavailability, as shown in CIHH rats (Siques et al., 2014b; Brito et al., 2015).

Hence, the above considerations would better explain the mild HAPH with apparently almost no functional capacity repercussion. In fact, the TAPSE index of ventricle performance is good. The left ventricle does not seem to be affected, except for a low proportion of aortic dilation, which could also be explained by the hyperdynamic state of this model. The latter findings agree with a previous report regarding left ventricle changes under hypoxia (Richalet and Pichon, 2014).

Moreover, as mentioned, the correspondence analysis using both mPAP values depicted two distinctive patterns of associations with metabolic, biomedical and occupational factors. This tool, albeit is a descriptive method, has allowed to demonstrate the association of elevated mPAP with others variables not found in the regression model. In fact, >15 years of working in this model is strongly associated to elevated mPAP, and additionally, the association of insulin >20 with elevated mPAP further and consistently support what was previously found. The findings obtained with this correspondence analysis are of utmost interest, plausible and depict a sort of signature of this exposure model; however, its role in understanding the pathophysiological process of HAPH remains to be determined. Because this analysis only allows association patterns to be determined, its potential usefulness for screening or prediction will require further studies.

Therefore, the current study highlights a rather novel finding, as discussed previously, in which an association of insulin and ADMA with high mPAP and HAPH was found. Also, the years spent at altitude in this CIHH model might be considered. It is worth noting that some of the above-mentioned associated variables to elevated mPAP, have been previously found to individually contribute to PH and HAPH (Parameswaran et al., 2006; Wu et al., 2009; Zamanian et al., 2009; Kane et al., 2016; San Martin et al., 2017). Until now, their contribution as a whole to right cardiac circuit parameter values in subjects working in long-term CIHH had not been clearly determined.

Although this study design has some limitations, its use was considered necessary to evaluate right cardiac status during long-term intermittent work at high altitude. First, a cross-sectional design allows the determination of only association, status and the absence of changes, but no predictive factors or cause and effect. Moreover, a prospective study might be very difficult.

However, a cross-sectional study has the advantages of the ability to measure several variables, lower cost, and the identification of important points to be studied in a future longitudinal study. Second, the echocardiogram was performed at SL on day 2 of the resting period for logistic reasons, which could produce pulmonary pressures lower than at high altitude. Nevertheless, this study allowed important findings in this condition to be determined. As a whole, while the characteristics found in these subjects could eventually be assumed to result from the study design, the rigorous methodology used, the strict selection and exclusion criteria, the use of more accurate technology and the literature provided support the reasonable validity of the findings of this study in this population. Additionally, altitude differences may explain the elevated proportions of altered morphological and functional right cardiac parameters found in this study compared with those in a previous cross-sectional study (Brito et al., 2007). In fact, the latter study was conducted at 3,550 m, while the subjects in the current study worked at over 4,400 m.

## Conclusions

In summary, this study contributes to the knowledge of long-term CIHH working conditions at altitude, a rather unique biological situation of hypoxia exposure. Thus, it corroborates the persistence of AMS and lack of excessive erythrocytosis. However, this study highlights some novel findings: a high prevalence of HAPH, which is similar to that reported in CH, and with higher numbers of subjects with elevated mPAP and RVWT. In addition to determining the right circuit morphological and functional status, this study is the first, to our knowledge, to identify an association between increased cardiometabolic variables and others with elevated mPAP in CIHH. Therefore, these findings are likely to have important implications for defining the epidemiological and biological features of this model or prompting actions for public health.

## Author contributions

JB, PS, and RL: conceived of and designed the study, analyzed and interpreted the data, drafted the manuscript, critically revised important intellectual content in the manuscript, and provided overall supervision; RR, KF, NL, JH, and RB: performed data acquisition, analysis, or interpretation and critically revised important intellectual content in the manuscript; FL-V: assisted with critical decisions and revisions; JB, PS, RL, RR, FL-V, KF, NL, JH, and RB: contributed to the interpretation of results and critical revision of the manuscript, approved the final manuscript and agreed to be accountable for all aspects of the work.

### Conflict of interest statement

The authors declare that the research was conducted in the absence of any commercial or financial relationships that could be construed as a potential conflict of interest.

## Abbreviations

BP: Blood pressure
SBP: Systolic blood pressure
DBP: Diastolic blood pressure
SPAP: Systolic pulmonary artery pressure
mPAP: Mean pulmonary artery pressure
RVWT: Right ventricle wall thickness
FCRV: Four-chamber right ventricle
RVOT: Right ventricle outflow track
RAA: Right atrium area
PVR: Pulmonary vascular resistance
LVEF: Left ejection fraction
AD: Aortic diameter
TAPSE: Tricuspid annular plane systolic excursion
RV: Right ventricle
RVH: Right ventricle hypertrophy
ADMA: Asymmetric dimethylarginine
SDMA: Symmetric dimethylarginine
HP: Pulmonary hypertension
HAPH: High-altitude pulmonary hypertension
CIHH: Chronic intermittent hypobaric hypoxia
HPV: Hypoxic pulmonary vasoconstriction
WP: Waist perimeter
SL: Sea level.
